# Dentinogenesis imperfecta type II‐ genotype and phenotype analyses in three Danish families

**DOI:** 10.1002/mgg3.375

**Published:** 2018-03-06

**Authors:** Kawther Taleb, Eva Lauridsen, Jette Daugaard‐Jensen, Pekka Nieminen, Sven Kreiborg

**Affiliations:** ^1^ Department of Odontology, Pediatric Dentistry and Clinical Genetics Faculty of Health and Medical Sciences University of Copenhagen Denmark; ^2^ Resource Centre for Rare Oral Diseases Copenhagen University Hospital, Rigshospitalet Copenhagen Denmark; ^3^ Department of Oral and Maxillofacial Diseases, Clinicum University of Helsinki Helsinki Finland

**Keywords:** bioinformatics, dentinogenesis imperfecta, genetics

## Abstract

**Background:**

Dentinogenesis imperfecta (DI) is a rare debilitating hereditary disorder affecting dentin formation and causing loss of the overlying enamel. Clinically, DI sufferers have a discolored and weakened dentition with an increased risk of fracture.

The aims of this study were to assess genotype‐phenotype findings in three families with DI–II with special reference to mutations in the *DSPP* gene and clinical, histological, and imaging manifestations.

**Methods:**

Nine patients participated in the study (two from family A, four from family B, and three from family C). Buccal swab samples were collected from all participants and extracted for genomic DNA. Clinical and radiographic examinations had been performed longitudinally, and the dental status was documented using photographic images. Four extracted and decalcified tooth samples were prepared for histological analysis to assess dysplastic manifestations in the dentin. Optical coherence tomography (OCT) was applied to study the health of enamel tissue from in vivo images and the effect of the mutation on the function and structure of the *DSPP* gene was analyzed using bioinformatics software programs.

**Results:**

The direct DNA sequence analysis revealed three distinct mutations, one of which was a novel finding. The mutations caused dominant phenotypes presumably by interference with signal peptide processing and protein secretion. The clinical and radiographic disturbances in the permanent dentition indicated interfamilial variability in DI–II manifestations, however, no significant intrafamilial variability was observed.

**Conclusion:**

The different mutations in the *DSPP* gene were accompanied by distinct phenotypes. Enamel defects suggested deficit in preameloblast function during the early stages of amelogenesis.

## 1 INTRODUCTION

Dentinogenesis imperfecta (DI) is a rare hereditary disorder characterized primarily by defective dentin formation and resulting in early loss of the overlying enamel with high risk of tooth loss. DI has an estimated incidence of 1:8000 (Witkop, 1975) and can be classified into three subtypes: DI–I, DI–II, and DI–III (Shields, Bixler, & el‐Kafrawy, 1973). These subtypes can either appear in association with osteogenesis imperfecta (DI–I) or as an isolated finding associated with mutations in the dentin sialophosphoprotein gene *DSPP* (DI–II and DI–III) (Hart & Hart, 2009).

The *DSPP* gene contains five exons and four introns and encodes a precursor protein that is cleaved to form two mature proteins: dentin sialoprotein (DSP) and dentin phosphoprotein (DPP) (Bai et al., 2010). Exons 1–4 encode the DSP, while exon 5 encodes the carboxy terminus of the DSP and the entire DPP (Xiao et al., 2001; Zhang et al., 2007). The DSP and DPP are proteins that constitute major noncollagenous components in the dentin and have important functions in its mineralization. Studies with mutant mice have suggested that DSP regulates the initiation of dentin mineralization, while the DPP is associated with maturation of dentin mineralization (Suzuki et al., 2009).

DI–II (MIM 125490) is an autosomal anomaly of dental development with complete penetrance (Bixler, Conneally, & Christen, 1969; Malmgren, Lindskog, Elgadi, & Norgren, 2004). In the period 2001–2017 more than 40 different mutations in the *DSPP* gene have been identified (Bloch‐Zupan et al., 2016; Li et al., 2017; Liu et al., 2016). Clinically, DI–II is characterized by discolored deciduous and permanent teeth of which the primary dentition is often the most severely affected (Bixler et al., 1969). Teeth in patients with DI–II exhibit an opalescent hue, ranging from amber brown to grayish‐blue. Radiographically, the teeth have bulbous crowns with narrow roots, and a constriction at the cervix (Hart & Hart, 2007; Leal, Martins, Verli, de Souza, & Ramos‐Jorge, 2010). Pulpal obliteration occurs soon after eruption and sometimes even before eruption (Shields et al., 1973). Histologically, the dentin is characterized by a dysplastic appearance with reduced mineralization, irregular dentin tubules, and occasionally interglobular dentin (Barron, McDonnell, MacKie, & Dixon, 2008; Hart & Hart, 2007). Davis, Fearne, Sabel, and Norén (2015) found that although the pulp in teeth affected with D‐II appears to be completely obliterated, a network of interconnected vessels may remain, and they suggested that the presence of large dentinal tubules and the remnants of blood vessels can provide a pathway for bacteria from the oral cavity. The enamel has been reported to be normal in structure and mineral content (Malmgren, Lundberg, & Lindskog, 1998). However, Lee et al. (2013), Wang et al. (2011) and Bloch‐Zupan et al. (2016) reported the enamel to be hypoplastic. Furthermore, in teeth with DI–II, the supportive dentin layer is dysplastic and has been associated with a brittle enamel that is more prone to fracture (Finn, 1962). This exposes the underlying dentin and leads to severe attrition with risk of pulp exposure (Hart & Hart, 2007).

To the best of our knowledge, no previous studies have investigated the structure of enamel tissue in DI–II patients. In this study, we aimed to examine the dental phenotype, including the microstructure of the enamel and dentin, and discuss genotype‐phenotype findings in three affected families.

## 2 MATERIALS AND METHODS

### 2.1 Subjects

**Figure 1:**
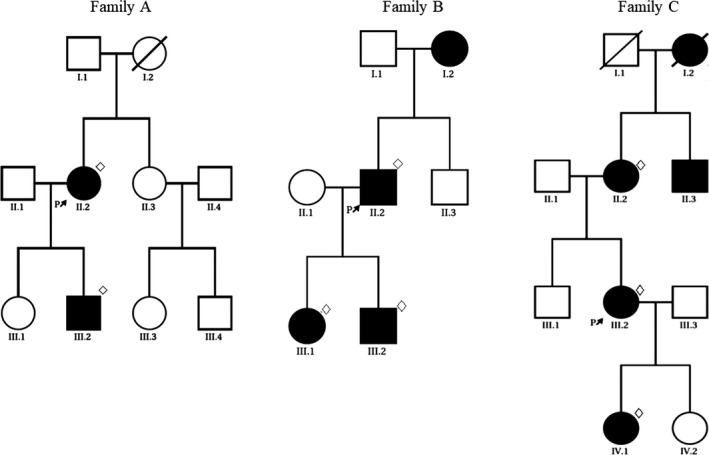
Pedigrees of the families A, B, and C. Unaffected individuals are shown as empty symbols and affected members are shown as solid symbols. Investigated patients are marked with a rhombus and the probands are marked with an arrow

During a 50‐year‐period, three Danish families from Zealand with DI–II were known to the Department of Odontology, Pediatric Dentistry and Clinical Genetics, Faculty of Health and Medical Sciences, University of Copenhagen, and the three probands had received extensive dental treatment. The family pedigrees are presented in Figure 1. A total of 9 individuals from these families were included in this study (two from Family A, four from Family B, and three from family C). However, Case B‐I.2 was not available for clinical examination, but participated in the genetic investigation. All patients were of Caucasian descent.

The distribution of the individuals according to gender and age is given in Table 1. Five of the 8 examined subjects had been seen for the first time prior to the age of three years, and another two had been examined prior to the age of 5 years. All subjects had been examined longitudinally.

**Table 1:** Age and gender distribution for the 8 examined patients and listing of available material in the individual patient

Patients photographs	Gender	Age at 1^st^ examination	Age at last examination	Dental x‐rays	Intraoral
A‐II.2	F	4.75	53	+	+
A‐III.2	M	2.50	17	+	+
B‐II.2	M	2.75	36	+	+
B‐III.1	F	2.75	16	+	+
B‐III.2	M	1.5	11	+	+
C‐II.2	F	11.5	18	−	+
C‐III.2	F	3.5	16	+	+
C‐IV.1	F	1.5	3	+	+

Informed written consent had been obtained from all participating patients or their legal guardians prior to examinations.

### 2.2 Methods

#### 2.2.1 Primer design, polymerase chain reaction, and DNA sequencing

Buccal swab samples were collected from all participants of the families. Two swabs were obtained from each patient from the left and right side of the buccal mucosa. The samples were subjected to mutational analysis at Department of Oral and Maxillofacial Diseases, Biomedicum, University of Helsinki, Finland.

As described in a previously published paper (Nieminen et al., 2011) the genomic DNA was isolated from buccal swab cells using Qiagen's kit (QiaAmp DNA mini kit) according to the manufacturer′s instructions (Qiagen, Chatsaworth, Calif., USA). PCR was used to amplify the immediate promoter and exons 1 through 5 with immediately flanking sequences of the *DSPP* gene (NM_014208.3). The PCR products were purified from primers and nucleotides with ExoSAP method and sequenced with ABI BigDye 3.1 reagent followed by capillary electrophoresis at the Institute for Molecular Medicine Finland Technology Center, Helsinki (for primer sequences and reaction conditions, see Table S1).

#### 2.2.2 Bioinformatics analysis of the mutations

Various software programs were used to investigate how or whether the identified mutations affected the function and structure of the *DSPP* gene.

PolyPhen‐2 (http://genetics.bwh.harvard.edu/pph2/) was used to predict the impact of the amino acid substitution on the protein structure and function in the missense mutations. Spliceport (http://spliceport.cbcb.umd.edu/), Netgene2 Server (http://www.cbs.dtu.dk/services/NetGene2/), and BGDP (http://www.fruitfly.org/seq_tools/splice.html) was used to predict effects on pre‐mRNA splicing. SignalP 4.1 (http://www.cbs.dtu.dk/services/SignalP/) was used to predict effects on the presence and location of signal peptide cleavage sites.

#### 2.2.3 Clinical and radiographic investigation

All investigated family members were examined both clinically and radiographically, except for Case B‐I.2 (see above) and Case C‐II.1 for whom only longitudinal intraoral photographs were available for study (Table 2). The clinical and radiographic examinations included assessment of tooth formation, maturation, eruption, discoloration, and attrition. In addition, assessment of periapical radiolucencies and the number of extracted teeth due to periapical conditions was carried out. Deviations in tooth formation were assessed as deviations in tooth number and malformations (e.g., cervical constriction and abnormally large or obliterated pulp chambers and root canals). In addition to the radiographic examination of teeth, a few extracted primary teeth were CBCT scanned and three dimensional (3D) images were produced to visualize the size of the pulp chamber. Dental maturity was scored according to the assessment system of Haavikko, and compared with available normative data (Haavikko, 1970). Discoloration of teeth was graded into: amber‐brown, light‐brown, yellow‐brown, and grayish‐blue. The degree of attrition was assessed from intraoral photographs according to *Broca′s* tooth wear index (Bardow, Pallesen, & Bakke, 2013).

**Table 2:** Clinical and radiographic dental findings in the affected and investigated members of the three families with DI

Family Patient	A III.2	A IV.2	B II.2	B III.1	B III.2	C II.1	C III.2	C IV.1^a^
Primary teeth								
Enamel defects	+	+	+	+ +	+	+	+	
Discoloration	AB	AB	AB	AB	AB	AB	AB	AB
Attrition (score)	4	4	4	3	4	3	4	4
Cervical constriction	+	+	+	+	+	NA	+	+
Obliterated pulp chambers	+	+	+	+	+	NA	+	−
Obliterated root canals	+	+	+	+	+	NA	+	−
Periapical radiolucencies	+	+	+	+	+	NA	NA	−
Extracted teeth due to periapical conditions	2	3	2	3	2	2	NA	0
Permanent teeth								
Enamel defects	+	+	+	+	+	+	+	+
Discoloration	YB	YB	LB	LB	LB	GB	GB	NA
Attrition (score)^b^	3	3	3	2	3	NA	2	NA
Cervical constriction	+	+	+	+	+	NA	+	NA
Obliterated pulp chambers	+	+	+	+	+	NA	+	NA
Obliterated root canals	+	+	+	+	+	NA	+	NA
Periapical radiolucencies	−	+	NA	+	−	NA	−	NA
Extracted teeth due to periapical conditions	1	1	2	1	0	NA	NA	NA

AB, amber brown; GB, grayish‐blue; LB, light‐brown; NA, not available; YB, yellow‐brown.

a The patient was only examined at the age of 3 years.

b Attrition patterns are based on *Broca′s tooth wear index*: a clinical measurement for tooth wear (Bardow et al., 2013).

All adult patients had received comprehensive dental treatment, including composite fillings, crowns, dental bridges or dental implants, prior to this study. A few affected subjects were still undergoing dental treatment.

#### 2.2.4 Histological investigation

Four tooth samples from Family B were available for histological investigation. The teeth were sent to Oral Pathology and Medicine, Department of Odontology, Faculty of Health and Medical Sciences, University of Copenhagen, over a 12‐year‐period. The teeth were fixed in 10% formalin and decalcified in ethylenediaminetetraacetic acid (EDTA). The teeth were embedded in paraffin, cut longitudinally, and stained with hematoxylin and eosin (HE). The stained sections were observed under the microscope. The dentin in each tooth was examined for: the appearance and branching of the dentin tubules, dentin mineralization, and the presence of areas void of dentin tubules.

#### 2.2.5 Tomographic imaging investigation

In a single subject, Case B‐III.2, the enamel in the permanent dentition was examined in vivo with optical coherence tomography (OCT) to make a qualitative and quantitative assessment of the tissue. An optical hand‐piece was placed on the tooth and its corresponding gingiva, and the tooth parts without dental restorations were scanned. Application and system setup have been described in a previously published paper (Hsieh et al., 2013).

## 3 RESULTS

### 3.1 Mutational analysis

**Figure 2:**
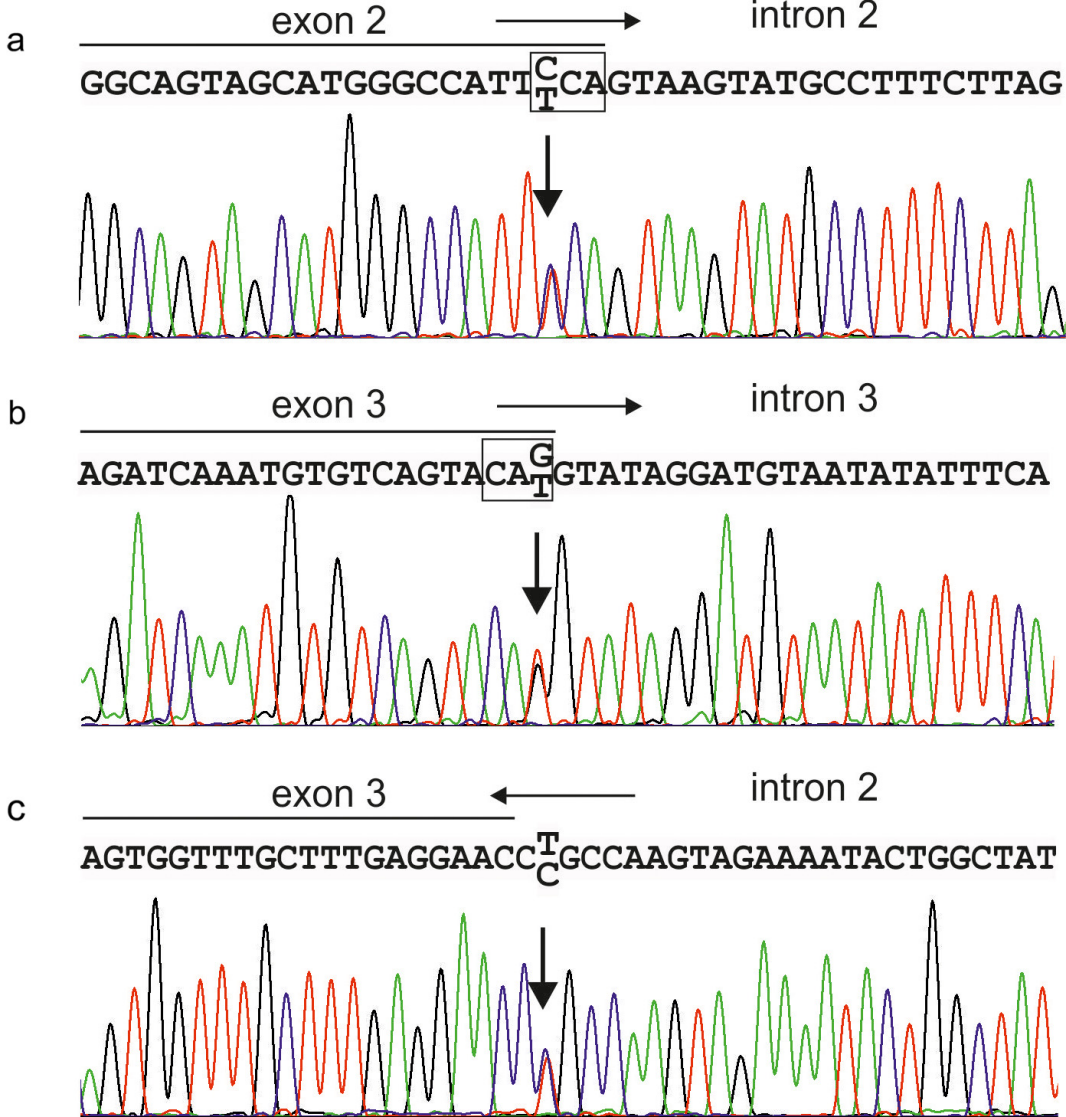
Mutational analysis. (a) A missense mutation involving a C→T transition at nucleotide 49 (p.Pro17Ser) was identified in Family A. (b) A novel missense mutation revealed a G→T transition at nucleotide 135 (p.Gln45His) in Family B. (c) A splice site mutation involving a A→G transition at c.52‐2 was identified in an acceptor splice site of intron 2 (c.52‐2 A>G) in Family C. Horizontal arrows indicate the direction of transcription. Boxes indicate the affected codons

Direct DNA sequence analysis revealed that all affected and investigated members of the three families had a heterozygous mutation in the *DSPP* gene. Mutation analysis of Family A revealed a missense mutation involving a C→T transition at nucleotide 49 in exon 2 (c.49C>T; Figure 2a). The transition causes a single amino acid substitution of a proline residue with a serine residue (p.Pro17Ser). A novel missense mutation was identified in Family B involving a G→T transition at nucleotide 135 in exon 3 (c.135G>T; Figure 2b), which results in an amino acid substitution of a glutamine residue by a histidine residue (p.Gln45His). Finally, mutation analysis of Family C revealed a splice site mutation involving an A→G transition at c.52‐2 in an acceptor splice site of intron 2 (c.52‐2 A>G; Figure 2c).

### 3.2 Bioinformatics findings

p.Pro17Ser and p.Gln45His impact on the protein structure and function were predicted as Probably Damaging (score: 0.998) by PolyPhen‐2 (see Table S2). For the normal sequence, the results of SignalP 4.1 suggested a most likely cleavage site was between position 15–16 and a max cleavage site probability of 0.724 (Table S2). However, the max cleavage site probability decreased to .600 at the same position for the substitution of p.Pro17Ser. Assuming excluding the whole exon 3, the same probability decreased to 0.622. Splicing prediction tools did not indicate a significant effect on the exon 2 donor site (junction of exon 2 and intron 2) by c.49C>T. However, they suggested loss of the exon 3 acceptor (junction of intron 2 and exon 3) and exon 3 donor (junction of exon 3 and intron 3) sites by the mutations c.52‐2A>G and c.135G>T, respectively (see Table S3), both of which have been associated with omission of exon 3 from the mature mRNA (Lee et al. 2011).

### 3.3 Clinical and radiographic findings

**Figure 3:**
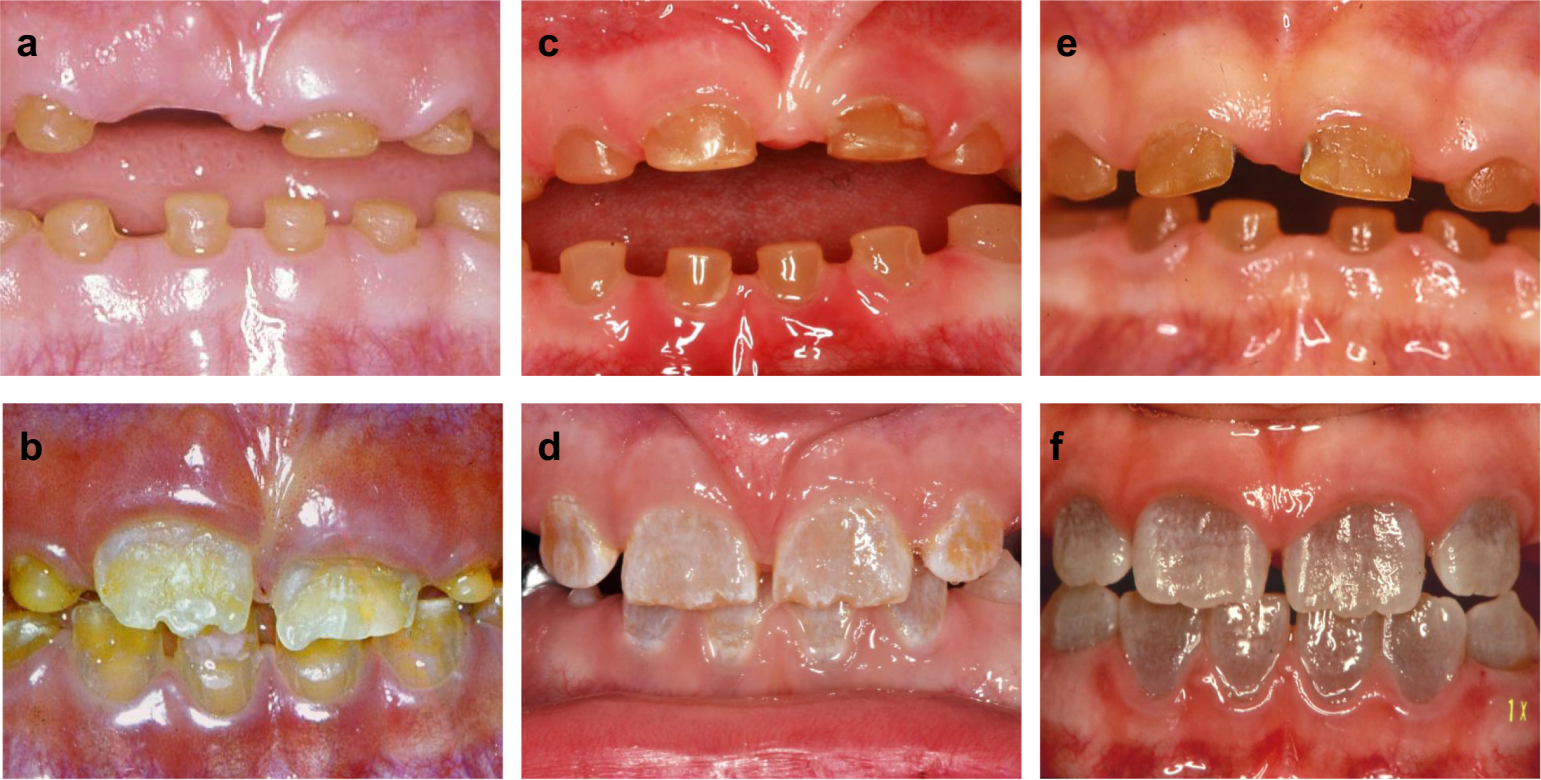
Clinical photographs of the primary and permanent teeth of the probands. Left row: Intraoral photographs of Case A‐II.2 (A: 6 years; B: 7½ years). Middle row: Intra oral photographs of Case B‐III.2 (C: 3½ years; D: 7½ years). Right row: Intraoral photographs of Case C‐III.2 (E: 3½ years; F: 9 years). Note the similar amber brown color of the primary teeth in the all cases (a–c), whereas there is a marked difference in color of the permanent teeth; in Case A the discolored teeth are yellow brown (b) Case B the discoloration is light‐brown (d), and in Case C it is grayish‐blue (f)

The clinical and radiographic dental findings in the 8 examined subjects are summarized in Table 2, and intraoral photographs of the representative proband from each family are shown in Figure 3.

#### 3.3.1 Primary dentition

**Figure 4:**
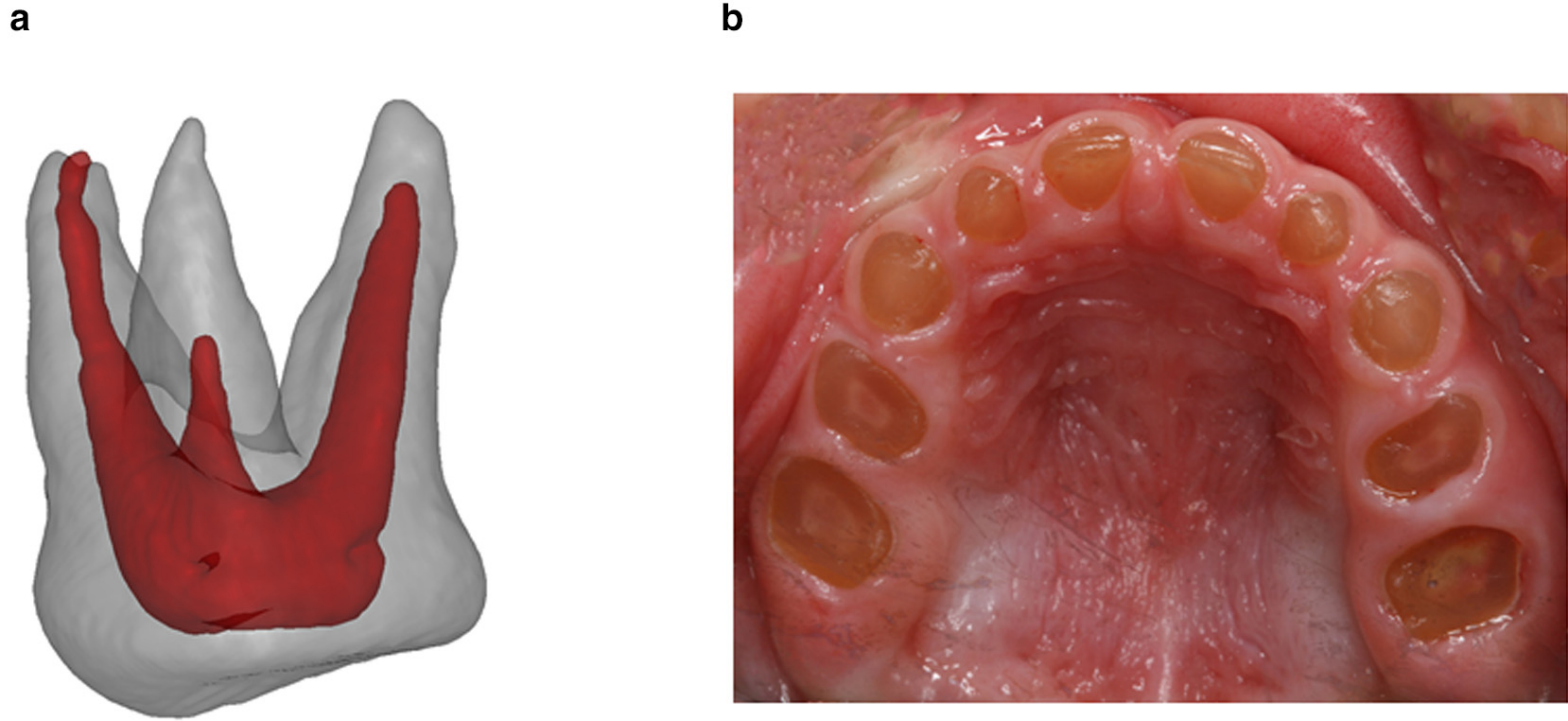
(a) 3D picture of an extracted maxillary first molar in the primary dentition of Case B‐III.2 at the age of 2 years. The size of the enlarged pulp chamber is illustrated. (b) Intraoral photograph of Case C‐IV.1 at the age of 2.5 years exemplifying the excessive attrition with pulp exposure in DI–II patients

The clinical and radiographic findings in the primary dentition showed limited variability both within and between the three families. In general, tooth morphology was characterized by cervical constriction. The pulp chambers and root canals were initially abnormally large, during tooth eruption, (see Figure 4a), but obliterated before the age of 5 years. The timing of dental maturity and eruption was unremarkable. The tooth crowns were, in general, amber brown and showed excessive attrition. In several cases attrition score 4 was seen already at 2.5–3 years of age (see Figure 4b). Periapical radiolucencies were observed in nearly all patients, and most patients had 2–3 teeth extracted due to periapical conditions.

#### 3.3.2 Permanent dentition

Tooth number was normal in all cases but one Case B‐III.2 had agenesis of the left mandibular second premolar. In general, the teeth were characterized by cervical constriction and obliteration of pulp chambers and root canals. Similar to the findings in the primary dentition, the pulp chambers and root canals were initially abnormally large, but soon after emergence the size of the pulp chambers and root canals decreased rapidly and soon obliterated completely. The timing of dental maturity and eruption was unremarkable. A qualitative clinical and radiographic assessment of the enamel gave the impression that was thin and hypoplastic in most cases and in all families. In addition, the teeth were discolored in all patients. The intrafamilial variability in tooth color was small. However, there was a marked interfamilial variability in tooth color: in Family A the teeth were yellow‐brown, in Family B the teeth were light‐brown, and in Family C they were grayish‐blue (see Figure 3). All permanent dentitions showed excessive attrition, but not nearly to the degree observed in the primary dentition. Periapical radiolucencies were observed in a few cases and a few teeth had been extracted due to periapical conditions, but not to the extent that was observed in the primary dentition.

### 3.4 Histological findings

The histological analysis of the teeth in Case B‐III.I revealed dysplastic changes in the dentin in all the examined teeth (Figure 4). The dentino‐enamel junction was characterized by lack of scalloping, although the underlying mantle dentin appeared normal in structure. Progressing inward to the circumpulpal dentin the pattern was, however, very atypical. The dysplastic appearance was characterized by abnormal granular dentin matrix due to interglobular mineralization and the irregular dentin tubules were reduced in number, arranged haphazardly, and of larger size.

### 3.5 Tomographic imaging findings

In vivo OCT images of the teeth in Case B‐III.2 revealed delineated areas of hypomineralization adjacent to the dentino‐enamel junction (Figure 6b).

## 4 DISCUSSION

This study identified three different mutations in the *DSPP* gene in the three DI–II affected families, one of which was novel. All mutations were localized to regions in which all previous DI–II mutations in the DSP coding part of the gene have been found. The different mutations in the *DSPP* gene possibly provide a molecular explanation to the distinct phenotypic features in the investigated families.

It is assumed that all missense, nonsense, and splice‐site *DSPP* mutations associated with exon 2 and 3 result in dominant phenotypes due to biochemical events that can interfere with signal peptide cleavage and effects on protein secretion (McKnight, Suzanne Hart, et al., 2008). The identified missense mutation (p.Pro17Ser) in Family A, was earlier reported in three affected DI–II families (Hart & Hart, 2007; McKnight, Simmer, Hart, Hart, & Fisher, 2008; Zhang et al., 2007) with similar phenotypic features as regards to enamel loss, attrition, discoloration and periapical radiolucency. The Pro17 residue was also mutated to a threonine (Pro17Thr) (Xiao et al., 2001) and leucine (Pro17Leu) (Li et al., 2012) residue in two different DI–II families. Pro17 is evolutionarily conserved in mammalian species, suggesting that it is critical for the function of the *DSPP* gene and thus a mutational hotspot (McKnight, Suzanne Hart, et al., 2008; Zhang et al., 2007). The exact molecular mechanism which caused DI–II is unclear. However, previous studies propose possible explanations (Rajpar et al., 2002; Xiao et al., 2001; Zhang et al., 2007). Pro17 is the second amino acid residue in the mature DSP protein. It falls within the conserved isoleucine‐proline‐valine (IPV) domain assumed necessary for efficient processing of the *DSPP′*s signal peptide (McKnight, Suzanne Hart, et al., 2008). The conservation of the chemical properties is therefore crucial for maintaining the biological functions of the protein (Li et al., 2012; Zhang et al., 2007). The hydrophobic proline is replaced with a hydrophilic serine altering the hydrophobic region and the identified mutation might therefore, interfere with signal peptide cleavage and lead to reduced translocation of DSP and DPP into the endoplasmic reticulum (ER) during translation (McKnight, Suzanne Hart, et al., 2008; Rajpar et al., 2002). This is in accordance with the results obtained from PolyPhen‐2, suggesting a *Probably Damaging* and SignalP suggesting defective signal peptide cleavage site recognition. The findings are supported by functional studies (Hart & Hart, 2007; Lee et al., 2013) indicating a retention of the Pro17 mutated protein in the ER and proposing a dominant negative effect due to cellular stress and reduction in protein export.

The identified novel missense mutation (p.Gln45His) in Family B affects the amino acid with a previously described nonsense mutation (Gln45stop) in a DI–II Chinese family (Zhang et al., 2001). The affected family had similar clinical manifestations of the disease, with special reference to attrition and discoloration, as observed in Family B in this study. The identified missense mutation is reported as *Probably Damaging* and according to Spliceport, Netgene2 Server, and BDGP the mutation may affect pre‐mRNA splicing in the donor splice site of exon 3. It has been shown that loss of this site causes a partial or complete skipping of exon 3 (Lee, Lee, Jung, Lee, & Kim, 2011) which would change the IPV motif to IPD. According to SignalP such change has a slight effect on signal peptide cleavage site recognition. Experimental evidence suggests that loss of exon 3 donor site also causes ER retention (Hart & Hart, 2007).

The identified splice site mutation in Family C (c.52‐2A>G) was previously reported in a Chinese DI–III patient (Li et al., 2017) with similar clinical features as regards discoloration, attrition, and enlarged pulps in the primary dentition, although data from the permanent dentition is not available due to the young age of the patient. The overlapping clinical findings are consistent with the DI–II and DI–III representing different phenotypic expression of the same disease, and not separate entities (Lee et al., 2009). Spliceport predicted that the mutation may affect recognition of exon 2 acceptor splice site shown to cause skipping of exon 3 and retention of the protein in the ER (Hart & Hart, 2007; Lee et al., 2011).

**Figure 5:**
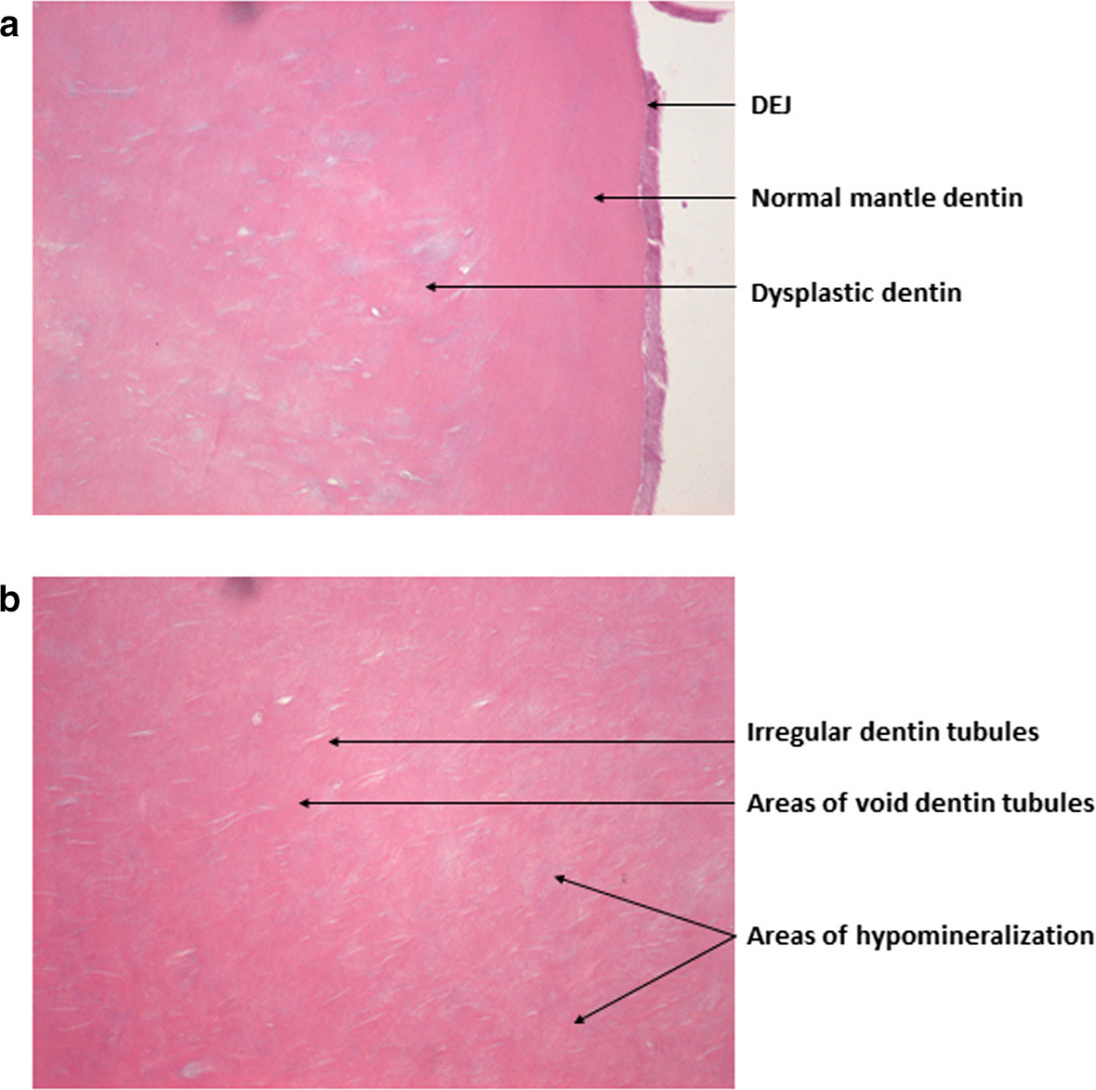
The histological analysis of the teeth in Case B‐III.1 revealed the presence of (a) A dentino‐enamel junction with an even appearance suggesting lack of scalloping, a normal mantle dentin, and an underlying layer of dysplastic dentin with incomplete mineralization, recognized as pale or blue staining areas. (b) The dentin tubules were irregular and enlarged with frequent branching in the circumpulpal dentin in the crown

**Figure 6:**
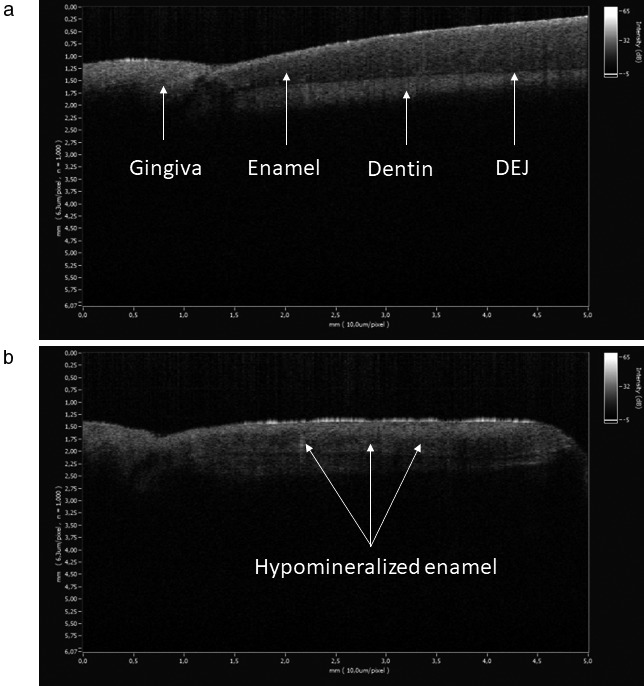
Imaging results of 2‐ from the author K.T and from Case B‐III.2. (a) The healthy enamel had a normal homogeneous appearance. (b) The dysplastic enamel was characterized by dark horizontally arranged areas of hypomineralization adjacent to the dentino‐enamel junction

The consequence of mutations in the *DSPP* gene is presumably defective formation of enamel and dentin, since *DSPP* is expressed in odontoblasts and transiently in preameloblasts (Bègue‐Kirn, Bartlett, & Butler, 1998). Enamel and dentin are formed as a result of various interactions between the ectoderm and ectomesenchyme, partly consisting of enamel formation on the dentin matrix (Thesleff, 2003). The enamel is in general of normal thickness in DI–II patients, but it fractures easily, and splits from the dentino‐enamel junction (Finn, 1962) suggesting a poor attachment of the enamel to the dentin either due to lack of scalloping at the dentino‐enamel junction, a poorly mineralized dentin, or a defect in the enamel (Finn, 1962; Lee et al., 2011). This study identified a dysplastic circumpulpal dentin, lack of scalloping of the dentino‐enamel junction (Figure 5), and a hypomineralized enamel of reduced strength (Figure 6b) proposing a deficiency of the epithelial structures as well in DI–II patients. Although wild‐type *Dspp*‐knockout mice had apparently normal enamel (Sreenath et al., 2003), it is possible that a severe form of mutant *DSPP* expressed transiently may disturb ameloblast differentiation and influence enamel formation (Lee, Lee, Hwang, et al., 2011).

Several similar phenotypic features were shared in the affected individuals, although interfamilial variability was observed in the permanent dentition (see Figure 3) as regards to discoloration. Clinically, the primary teeth were, in general, more severely affected than the permanent teeth. A possible explanation is that in primary teeth, the enamel is thinner and can expose the discolored dentin very early, leading to extreme tooth wear (Malmgren et al., 2004). Obliteration of pulps and root canals could be a consequence of the attrition. However, pulpal obliteration can also occur before tooth eruption which indicates that the phenomenon is a rather a manifestation of the disease (Finn, 1962). Although the teeth seemed completely obliterated radiographically, a network of interconnected vessels may remain, providing a pathway for bacteria from the oral cavity (Davis et al., 2015). This may account for the presence of pathologic periapical conditions despite obliterated pulps in both dentitions.

In general, early diagnosis is of considerable importance for the treatment of DI–II in order to obtain a favorable prognosis. Although the treatment of the investigated patients varied, all treatments aimed to ensure removal of infectious sites and/or prevention of pain, establishing a good occlusion by preventing loss of vertical facial dimension, and restoring aesthetics (Barron et al., 2008; Leal et al., 2010; Sapir & Shapira, 2001).

## 5 CONCLUSION

In summary, the enamel and dentin were examined, and the described DI–II families were assessed clinically, radiographically, and histologically. Three distinct mutations were identified in the DSP region of the *DSPP* gene, one of which was novel. The molecular pathogenesis by which the *DSPP* mutations have caused DI–II was characterized in order to elucidate the genotype‐phenotype correlation. The present data indicate that DI–II might affect both dentin and enamel. However, further studies are needed to elucidate the underlying mechanisms of enamel defects in DI–II patients.

## CONFLICT OF INTEREST

None declared.
