# Supplementary material for: Dentinogenesis imperfecta type II‐ genotype and phenotype analyses in three Danish families
Source: Mol Genet Genomic Med. 2018 Mar 6;6(3):339–49. doi: 10.1002/mgg3.375 (PMC6014476; doi:10.1002/mgg3.375)
Supplement: Supplementary file 1 [file MGG3-6-339-s001.docx]

**Supporting Table 1.** Primers and PCR conditions for the mutational analysis of *DSPP*

| target |  | primers | bp | PCR | add | seq^**)^ | add |
| --- | --- | --- | --- | --- | --- | --- | --- |
|  |  |  |  | ann T x cycles^*)^ |  | ann T |  |
|  |  |  |  |  |  |  |  |
| promoter | F | GACGGTCAAGTTTTTGAATGC | 606 | 58 x 32 |  | 58 |  |
|  | R | GCTCCTATGAGGCTGTTTCAC |  |  |  |  |  |
|  |  |  |  |  |  |  |  |
| exon 1 | F | CAAGAAATGTCAGGGTGACAGA | 479 | 55 x 30 |  | 55 |  |
|  | R | AAATCAAACTGGCTTCATCTTTG |  |  |  |  |  |
|  |  |  |  |  |  |  |  |
| exon 2 | F | TGTCCAGGAAAAGGGCAAAT | 213 | 55 x 30 |  | 55 |  |
|  | R | AAGTGAAGAGGTTTTCTAAGAAAGG |  |  |  |  |  |
|  |  |  |  |  |  |  |  |
| exon 4a | F | CACATATTCACAAATAAGAACCTT | 405 | 55 x 30 |  | 55 |  |
|  | R | CCCTCCTACTTCTGCCCACT |  |  |  |  |  |
|  |  |  |  |  |  |  |  |
| exon 4b | F | CCTGGTGCATGAAGGTGATA | 545 | 55 x 30 |  | 55 |  |
|  | R | TATCCAGGCCAGCATCTTCT |  |  |  |  |  |
|  |  |  |  |  |  |  |  |
| exon 4c | F | ACCTCAGATCAACAGCAAGAG | 544 | 65x2,63x2,61x33 | ad 3 mM MgCl_2_ | 60 | 1 M betaine |
|  | R | CATCAATAGATGGAGGGGAAGA |  |  |  |  |  |
|  |  |  |  |  |  |  |  |
| exon 5a | F | CCTATGGCAACTTTTCCCAGT | 443 | 55 x 30 |  | 55 |  |
|  | R | TCTCCTCGGCTACTGCTGTT |  |  |  |  |  |
|  |  |  |  |  |  |  |  |
| exon 5b | F | GCCAAAAATCAGAACCAGGA | 594 | 58 x 32 |  |  |  |
|  | R | CTGACTTGCTGTCACTGCTG |  |  |  |  |  |
|  |  |  |  |  |  |  |  |
| exon 5c^***)^ ^F^ | | CAAAAGGAGCAGAAGATGATGA | 2518 | 58 x 32 | 3 % DMSO | 58 |  |
|  | R | TCCCCCAGTTGTTTTTGTTT |  |  |  |  |  |
| exon 5e | F | CAGCAAATCAGAGAGCGACA | 797 | 61x35 | 2 % DMSO | 60 | 1 M betaine |
|  | R | TGCTATTGCTGCTTTCGTTG |  |  |  |  |  |
|  |  |  |  |  |  |  |  |
| exon 5f | F | GATAGCGACAGCAGCAATAGAA | 1250 | 67x2,66x2,64x35 | 2 % DMSO | 60 | 1 M betaine |
|  | R | CTTGCTCTGGCTGTCACTCTCA |  |  |  |  |  |
|  |  |  |  |  |  |  |  |

^*)^All PCR reactions were performed as hot start, i.e. adding the polymerase only after warming the reaction to 94 degrees_._

^**)^All sequencing reactions were performed with 25 cycles.

^***)^Amplimer for exon 5c spans over both 5e and 5f.

**Supporting Table 2**. Bioinformatics analysis results: prediction of the mutations effect on the protein structure and function.

| Family | Protein | cDNA | Location | PolyPhen-2 | | SignalP 4.1 | |
| --- | --- | --- | --- | --- | --- | --- | --- |
|  |  |  |  | Prediction | Score | Most likely cleavage site | Max cleavage site probability |
|  | normal |  |  |  |  | Between pos.15 and 16 | 0,724 |
| A | Pro17Ser | c.49C>T | Exon 2 | Probably Damaging | 0.998 | Between pos.15 and 16 | 0,600 |
| B | Gln45His | c.135G>T | Exon 3 | Probably Damaging | 0.998 | Between pos.15 and 16 | 0,622*) |
| C |  | c.52-2A>G | Intron 2 |  |  | Between pos.15 and 16 | 0,622*) |

*) Assuming exon 3 skipping

| Family | Protein | cDNA | Location | exon 2 donor | | | exon 3 acceptor | | | | exon 3 donor | | |
| --- | --- | --- | --- | --- | --- | --- | --- | --- | --- | --- | --- | --- | --- |
|  |  |  |  | Spliceport | Netgene2 | BDGP | Spliceport | Netgene2 | BDGP | Spliceport | | Netgene2 | BDGP |
|  | normal |  |  | 0.904 | 0,54 | 0,98 | 0,906 | 0,25 | 0,97 | 0,939 | | 0,35 | 0,89 |
| A | Pro17Ser | c.49C>T | Exon 2 | 0,806 | 0,52 | 0,98 | 0,906 | 0,25 | 0,97 | 0,939 | | 0,35 | 0,89 |
| B | Gln45His | c.135G>T | Exon 3 | 0.904 | 0,54 | 0,98 | 0,906 | 0,25 | 0,97 | not detected | | not detected | not detected |
| C |  | c.52-2A>G | Intron 2 | 0.904 | 0,54 | 0,98 | not detected | not detected | not detected | 0,939 | | 0,34 | 0,89 |

**Supporting Table 3**. Bioinformatics analysis results: Splice site prediction.
